# *Fragaria vesca* L. Extract: A Promising Cosmetic Ingredient with Antioxidant Properties

**DOI:** 10.3390/antiox9020154

**Published:** 2020-02-14

**Authors:** Joana Couto, Artur Figueirinha, Maria Teresa Batista, António Paranhos, Carla Nunes, Lídia Maria Gonçalves, Joana Marto, Manuel Fitas, Pedro Pinto, Helena Margarida Ribeiro, Maria Eugénia Pina

**Affiliations:** 1 Faculty of Pharmacy of University of Coimbra, University of Coimbra, 3000-548 Coimbra, Portugal; joana.jpoc92@gmail.com (J.C.); amfigueirinha@ff.uc.pt (A.F.); mtpmb@ff.uc.pt (M.T.B.); topar@ff.uc.pt (A.P.); carlapumky@gmail.com (C.N.); 2 LAQV, REQUIMTE, Faculty of Pharmacy, University of Coimbra, 3000-548 Coimbra, Portugal; 3 Center for Pharmaceutical Studies, Faculty of Pharmacy, University of Coimbra, 3000-548 Coimbra, Portugal; 4 CIEPQPF, Research Center for Chemical Processes Engineering and Forest Products, University of Coimbra, 3030-790 Coimbra, Portugal; 5 Center for Neuroscience and Cell Biology, University of Coimbra, 3004-517 Coimbra, Portugal; 6 Research Institute for Medicines (iMed.ULisboa), Faculty of Pharmacy, Universidade de Lisboa, 1649-003 Lisbon, Portugal; lgoncalves@ff.ulisboa.pt (L.M.G.); geral@phdtrials.com (P.P.); hribeiro@campus.ul.pt (H.M.R.); 7 PhD Trials, Avenida Maria Helena Vieira da Silva, nº 24 A, 1750-182 Lisbon, Portugal; mfitas@phdtrials.com

**Keywords:** *Fragaria vesca* L., antioxidant properties, hydrogel, topical application, cutaneous compatibility

## Abstract

*Fragaria vesca* L. (*F. vesca*), popularly known as wild strawberry, is a plant from the Rosaceae family, found in temperate and subtropical areas of the northern hemisphere. *F. vesca* leaves have been shown to have antiseptic, emollient, and dermatological protection properties, due to the presence of bioactive compounds, such as flavonoids, phenolic acids, ellagitannins, and proanthocyanidins. In this study, a *F. vesca* extract was obtained by an optimized extraction process, and was characterized by HPLC, ROS scavenging activity, cytotoxicity assays in HaCaT cells, and tyrosinase inhibitory activity determination. The most active extract was then incorporated in a hydrogel with hydroxyethylcellulose at 2% (*w/w*), which was characterized at the physicochemical, stability, cytotoxicity, and ROS scavenging activity levels to evaluate its quality, safety, and efficacy. In vivo studies, human repeat insult patch testing, and an assay to determine their antioxidant efficacy, were also performed. The results showed that the *Fragaria vesca* extracts had antioxidant activity and that the *F. vesca* extract-based hydrogel exhibited cutaneous compatibility, acceptability and antioxidant efficacy, being stable, and suitable for topical application.

## 1. Introduction

According to the World Health Organization (WHO), 65% of the world’s population has incorporated medicinal plants into their primary health care (Fabricant and Farnsworth, 2001). Plants provide an unlimited source of novel and complex chemical structures produced by secondary metabolism, that are responsible for their biological activity. The structural diversity of these phytoconstituents make them unlimited sources of new active compounds [1,2]. A particularly important group of secondary metabolites are the phenolic compounds. There are over 10,000 known structures, performing a wide range of bioactivities, namely antioxidant, anti-inflammatory, antimicrobial, and anticancer. Their analysis and characteristics are indicative of their great diversity in nature [3].

*Fragaria vesca*, wild strawberry, is a rich source of biologically active phenolic compounds such as tannins, anthocyanins, flavonoids, and phenolic acids [4]. Anti-inflammatory, anticoagulant, vasodilatory, and antioxidant effects are some of the reported activities [5,6]. *F. vesca* leaves contain flavonoids, proanthocyanidins, ellagitannins, phenolic acids, volatile oils, catechins, methyl salicylate, ellagic acid, borneol and also trace amounts of alkaloids, with ellagitannins being the main phenolic compound [5,6,7,8,9]. Due to the presence of a great diversity of polyphenolic compounds and their antioxidant properties, *F. vesca* leaves provide a protective action on the skin [10]. Moreover, hydrolyzable tannins, namely the ellagitannins have antioxidant and antimelanogenic activities, through a mechanism involving the decrease in the expression of tyrosinase, an essential enzyme catalyzing the first steps of endogenous melanin production [11,12]. Ellagic acid, produced from the hydrolysis of ellagitannins, has also been reported to suppress melanogenesis [11,13]. The depigmenting effect seems to be related to the tyrosinase antioxidant capacity [12]. Another suggested possible mechanism is the inhibition of tyrosinase activity by ellagic acid, which regulates melanin production at the basal epidermal layer by quenching copper ions at the active site [13,14].

The aim of this work was to obtain a cosmetic formulation containing extract of *F. vesca* leaves, with enhanced effect on the skin, namely with antioxidant properties, and showing cutaneous acceptability and compatibility.

## 2. Materials and Methods

### 2.1. Materials

*Fragaria vesca* leaves were acquired from Granja de Figueira do Lorvão, Penacova, Portugal, on May 2011. Ethanol, acetonitrile, formic acid, sodium carbonate, and Folin Ciocalteu reagent were obtained from Merck (Lisboa, Portugal). Tetrahydrofuran (THF) was obtained from Koch-Light (Johannesburg, Gauteng, South Africa); iron (III) chloride hexahydrate 98% from Acros (Queluz, Portugal). HPLC methanol, came from Merck (Lisboa, Portugal). Hydroxyethyl cellulose (HEC) was obtained from Ashland (Wilmington, DE, USA). l-DOPA (0.5 mM), mushroom tyrosinase, pyrogallol, and 3-(4,5-dimethylthiazol-2-yl)-2,5-diphenyl-tetrazolium bromide (MTT) were obtained from Sigma-Aldrich (St. Louis, MO, USA). Dulbecco’s modified eagle medium (DMEM) with stable glutamine and foetal bovine serum (FBS) were purchased from Bioconcept (Allschwil, Switzerland).

### 2.2. Methods

#### 2.2.1. *Fragaria vesca* Extract Preparation

Dried *F. vesca* leaves were powdered, sieved (ENDECOTTS sieve, 2500 mesh/ cm^2^), and extracted with 50% aqueous ethanol (1:10 *w/v*), at room temperature, with magnetic stirring for 1 h at 750 rpm. Three extractions were carried out, until no ellagitannins were detected by TLC. The extract was then filtered under vacuum and ethanol removed with a rotary evaporator. The obtained aqueous extract was lyophilized and stored at −22 °C. For TLC, silica-gel plates were used and a mobile phase constituted by water:acetonitrile:THF:formic acid (80:5:15:2, *v/v/v/v*). For ellagitannins detection, the plates were sprayed with 3% iron (III) chloride solution in methanol (*w/v*) [15].

#### 2.2.2. High-Performance Liquid Chromatography (HPLC)

HPLC-PDA analysis was performed on a Gilson chromatograph equipped with two pumps (models 305 and 306, Gilson, Middleton, WI, USA); mixer (model 811, Gilson, Middleton, WI, USA); manometer module (model 805, Gilson, Middleton, WI, USA); automatic injector (Gilson 234, auto injector, Gilson, Middleton, WI, USA) coupled to a photodiode detector (PDA) (Gilson, model 107, Gilson, Middleton, WI, USA); and a Unipoint system data control and processing station (Unipoint 2.10, Gilson, Middleton, WI, USA).

Separation occurred in a RP18 Spherisorb Waters ODS-2 analytical column, particle size 5 μm (4.6 × 250 mm, Milford, MA, USA), and a precolumn KS 30/4 Nucleosil 120-5 C-18, Macherey-Nagel GmbH & Co. KG (Düren, Germany). The mobile phase consisted of 5% formic acid (*v/v*) (eluent A) and methanol (eluent B), following a gradient profile with the variations: 0–10 min (5–15% B), 10–15 min (15–25% B), 15–50 min (25–50% B), 50–60 min (50–80% B), 60–70 min (80–100% B). An aliquot of 100 μL from 1.8 mg extract solubilized in 1 mL methanol 50% (*v/v*) was injected at the flow rate of 1 mL/min, at 21 °C.

HPLC-PDA-ESI/MS^n^ structural identification of compounds present in *Fragaria vesca* extract was carried out on a liquid chromatograph with a photodiode spectrophotometer—PDA detector (Thermo Finnigan Surveyor, San Diego, CA, USA) interfaced with a linear ion trap mass spectrometer (LIT-MS) (LTQ XL, Thermo Scientific, Waltham, MA, USA). The sample was injected on a Spherisorb ODS-2 column (150 × 2.1 mm id; particle size, 3 μm; Waters Corp., Milford, MA, USA) with a Spherisorb ODS-2 guard cartridge (10 × 4.6 mm id; particle size, 5 μm; Waters Corp., Milford, MA, USA) at 25 °C. The elution was performed using 1% aqueous formic acid (*v/v*) (A) and methanol (B) as mobile phase, with a gradient profile of 0–10 min (5%–15% B), 10–15 min (15%–25% B), 15–50 min (25%– 50% B), 50–60 min (50–80% B), 60–70 min (80%–100% B), and an isocratic elution for 5 min, at a flow rate of 200 μLmin^−1^. The PDA detection was recorded in a wavelength range of 200–450 nm, followed by the detection in the mass spectrometer. Mass spectra were acquired in a negative ion mode. The mass spectrometer performed three consecutive scans: Full mass (*m/z* 125–2000), MS^2^ of the most abundant ion in the full mass, and MS^3^ of the most abundant ion in the MS^2^. Source and capillary voltage were 4.7 kV and −7 V, respectively. Capillary temperature was 275 °C. Nitrogen was used as sheath and auxiliary gas at 20 and 7 Finnigan arbitrary units, respectively, and helium as collision gas with a normalized energy of 49%. Data treatment was carried out with the XCALIBUR software (Thermo Scientific, Waltham, MA, USA).

#### 2.2.3. Tyrosinase Inhibition

The assay was carried out using l-DOPA as substrate, and performed according to a previously described method [16], with slight modifications. The reaction mixture contained a sodium phosphate buffer (100 mM, pH 6.8) with or without test sample, mushroom tyrosinase (35 units), and l-DOPA (0.5 mM) in a final volume of 1 mL. The mixture was preincubated at 25 °C for 10 min before adding substrate to start the reaction. Dopachrome formation was monitored by measuring the absorbance at 475 nm every 10 s for at least 3 min. The percentage of tyrosine’s inhibition of activity was calculated as follows:

(1) $$Inhibition\;\left(\%\right)=\frac{\left(A-B\right)}{A}\times 100$$

where, *A* and *B* represent the difference in the absorbance of the control and test samples, respectively, between incubation times of 0.5 and 1.0 min.

#### 2.2.4. Preparation of Topical Formulation: *F. vesca* Based Hydrogel

The composition of the formulation is listed in Table 1. All components were weighed on a semi-micro balance OHAUS DV215CD Discovery (Ohaus, Nänikon, Switzerland). Afterward, the HEC was transferred to a porcelain mortar; the extract was incorporated into the water, as well as ethanol. This mixture was added with stirring to the HEC in the mortar, three times at 10 min intervals, until homogenization. The formulation was packed and allowed to equilibrate for 24 h prior to use in subsequent studies.

#### 2.2.5. Physicochemical Characterization and Stability of Topical Formulations

The organoleptic characteristics of the formulation were evaluated, namely, the color, appearance, odor, and pH which, for skin is recommended to be in the 4.5–6.5 range [17,18]. The pH of the formulations were determined with a digital Consort pH meter C3010 (Dias de Sousa Portugal, Alcochete, Portugal), precalibrated with standard buffer solutions (pH 4.00, 7.00, and 10.01). One gram of hydrogel sample was diluted in 10 mL of distilled water and stirred on a magnetic plate until complete dissolution. The results are presented as the average of three measurements.

A Viscostar Plus viscometer was used to measure viscosity. The spindle 6 probe was used, at 30 rpm speed at room temperature (20–25 °C). The results presented are expressed in mPa s, as the average of three measurements.

Texture assays were performed in a Dias de Sousa TA-XT Plus texturometer (Stable Micro Systems Ltd., Surrey, UK). The samples were placed in cylindrical tubes, avoiding the introduction of air bubbles. An analytical probe P10 was used; it was twice depressed in the sample, at a defined speed (5 mm/s), with a recovery period of 8 s between consecutive compressions. From the force-time graph obtained, the following mechanical parameters were determined: Hardness (H)—given by the maximum peak of the first compression; compressibility—calculated from the area under curve 1 (AUC_1_); adhesiveness—calculated from the AUC_2_, corresponding to the negative part of the first cycle of compression; cohesion—equivalent to the ratio AUC_3_/AUC_1_; elasticity—corresponding to the ratio of the time required to achieve maximum structural deformation on the second compression cycle to that on the first compression cycle [19,20]. Texture measurements were performed at room temperature (20–25 ° C).

To evaluate the stability of the developed formulation, the following parameters were determined: Color, appearance, odor, pH, viscosity, and texture analysis, for 28 days (on the day of preparation (day 0), day 7, and day 28 after preparation). The hydrogel was divided into four samples, stored in different conditions in glass containers: One sample was stored at room temperature, influenced by solar light (S1); a second sample stored at room temperature in the dark (S2); a third sample stored in the refrigerator, with temperatures ranging from 5 to 9 °C (S3); and a fourth sample, stored in a greenhouse, at 40 °C with 75% relative humidity (RH) (S4).

#### 2.2.6. Total Tannins Content

The total tannins were determined in the extract and in the final hydrogel formulation. The method used was described in the European Pharmacopoeia 9th Edition [21]. For determination of tannin content in the hydrogel, the procedure to determine A1, A2, and A3 was repeated, using an amount of hydrogel equivalent to the extract from the corresponding assay. The content of tannins was expressed as % (*w/w*) of pyrogallol.

#### 2.2.7. In Vitro Cell Culture Assays

The human keratinocyte cell line HaCaT (CLS, Eppelheim, Germany) was used to evaluate the in vitro cytotoxicity and the ROS scavenging activity of the extract and the hydrogel formulation. The cells were routinely cultured in 75 cm^2^ culture flasks containing DMEM supplemented with 10% (*v/v*) FBS, at 37 °C in a humidified atmosphere of 5% CO_2_. Cells were subcultured at 80% of confluence.

##### Cytotoxicity Studies

The cell viability was assessed with the MTT assay, which is based on the reduction of the dye MTT to formazan by cellular dehydrogenases [22]. Briefly, HaCaT subconfluent cells grown in 24-well plates were treated with several concentrations of extract (0.1 to 2 mg/mL) or of the hydrogel formulation (1 to 5 mg/mL). After 24 h, the culture medium was removed, and cells were washed with phosphate-buffered saline (PBS). MTT was added to each well at the final concentration of 0.5 mg/mL. After 60 min of incubation at 37 °C in a humidified atmosphere of 5% CO_2_, the MTT was removed and formazan crystals were dissolved by the addition of 0.5 mL of DMSO. The extent of MTT reduction was evaluated spectrophotometrically, at 540 nm in a Synergy HT microplate reader. Results were expressed as a percentage of the control cells, i.e., nontreated cells.

##### Reactive Oxygen Species (ROS) Production Measurement

The effect of different concentrations of sample on the reactive oxygen species (ROS) production, induced by 500 μM hydrogen peroxide or by UVB radiation, in HaCaT cells, was evaluated by a fluorimetric method with the probe 2′,7-dichlorodihydrofluorescein diacetate (H2-DCFDA), as described previously [22].

#### 2.2.8. In Vivo Safety and Efficacy Tests

These studies intended to assess the safety and the antioxidant efficacy of a cosmetic product, after application under the normal conditions of use.

##### Simple Patch Test

The simple patch test was performed on 10 volunteers, aged between 18 and 65 years, with skin photo types between I and IV, who were informed about the procedure and had signed the informed written consent. Volunteers with dermatological or other medical or physical problems, and pregnant and nursing women were excluded from the study.

The hydrogel was applied once in an amount of 20 μL, and was in contact with the skin for 48 h, protected by an adhesive which facilitated the passage of the components through the skin. The experimental area of application was examined at 15 min, 24 and 48 h after removal of the adhesive. The security test followed the Protocol 01_01 with the Ethical Comission Opinion 006/2014 from 2 September, 2014.

##### Human Repeat Insult Patch Test (HRIPT)

This study was performed according to the Marzully and Maibach HRIPT protocol. It lasted six weeks, including an induction phase of three weeks, two weeks of rest, and the final week of the challenge. The experimental chosen area for the application was the back. In the induction phase, 20 μL of the product was applied and protected by an occlusive adhesive. It was kept in contact with the skin for 48 or 72 h over the weekend, after which the adhesive was removed and the skin reaction was evaluated. The application was repeated nine times in the initial phase of three weeks. The remaining period lasted two weeks, during which no product was applied. In the challenge phase, the product was reapplied in the same place of the induction phase and in a complementary place where there was no previous application. Removal was done after 48 h and the skin reaction was evaluated at 48, 72, and 96 h after application. For this study, 50 healthy volunteers were chosen, who were informed about the procedure and had signed the informed consent. The same specific noninclusion criteria of the simple patch test was applied. The Ethical approval code is 006/2014 (date: 02/09/2014, project identification code: 5671216.E).

##### Antioxidant Efficacy

Skin color was determined using a tristimulus color analyzer that measures the reflected color. A Minolta chromameter CR-400 (Minolta, Tokyo, Japan) was used to detect any slight deviation in the xenon’s light spectral distribution. The system provides data for the luminance (L*), a* (red-green) and b* (blue-yellow) color distribution. At D1, one arm was treated with the investigational product, while the other arm was untreated and used as the control. At D1, a solution of β-carotene was applied in both arms. The color was measured before and after UVA irradiation. β-carotene is a yellow chromophore molecule that when oxidized loses its chromophore capability and color. This discoloration can be monitored by colorimetry (b* parameter). The same area was evaluated on the first day (D0) and then after 28 days (D28) using the same procedure. Between D0 and D28, the volunteers applied the topical products in the areas indicated by the principal investigator.

#### 2.2.9. Statistical Analysis

All results of pH, viscosity, and absorbance measurements were subjected to the 5% confidence test for Grubbs’ outlier’s elimination, using Microsoft Excel. Statistical analysis of cell viability results was performed using ANOVA in Microsoft Excel (Microsoft, Redmond, WA, USA).

## 3. Results

### 3.1. Characterization of the Extract

**Figure 1:**
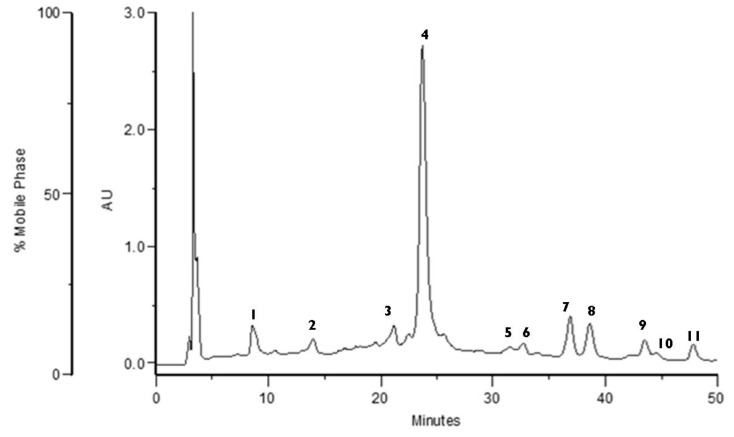
Chromatographic profile obtained with HPLC-PDA of *Fragaria vesca* 50% aqueous ethanol extract (280 nm).

The chromatogram obtained by HPLC at 280 nm of the *F. vesca* 50% aqueous ethanol extract is shown in Figure 1. The polyphenol profile was obtained through the retention times, elution order, and UV spectra, in comparison with previously obtained chromatographic profiles [5]. HPLC-PDA-ESI/MSn structural identification of the main polyphenols was inferred from Liberal et al. [5].

Through UV spectra the following compounds were identified: Proanthocyanidins (1, 2), a phenolic acid (3), ellagitannin (4), flavonoids (5, 7, 8, 10), and ellagic acid derivatives (6, 9, 11).

Peaks 1 and 2 were identified essentially as proanthocyanidins because they exhibited UV maxima and spectral profiles characteristic of flavan-3-ols. UV spectra with maxima near 250 and 324 nm and a shoulder at 298 nm indicate that peak 3 could be a caffeic acid derivative. This assumption is corroborated by its mass spectrum, which exhibits a molecular ion at *m/z* 353 and a base peak at *m/z* 173 in MS^2^, fragmentation pattern that suggests a 4-*O*-caffeoyl-quinic acid [23].

The major compound in the extract (peak 4) presented a molecular ion at *m/z* 1869, losing two HHDP units (302 amu) and originating fragments at *m/z* 1567 and 1265 in the MS^2^. Further losses of a glucosyl group (162 amu), gallic acid (170 amu), and another HHDP group (302 amu) originated signals at *m/z* 1103, 933, and 631, respectively. In the MS^3^, the fragment at *m/z* 1567 originated a signal at *m/z* 935 from the loss of a galloyl-HHDP glucose moiety. Thus, peak 4 was tentatively identified as the ellagitannin agrimoniin or sanguiin H-6, which are two isomeric forms. However, the presence of agrimoniin as the main ellagitannin present in *F. vesca* and *Fragaria ananassa* is consistent with previous studies [24].

Peaks 7 and 10 have the same UV spectra profiles, presenting an absorption maximum at 346 nm and MS spectra with the same aglycone fragment, at *m/z* 285, that is characteristic of the kaempferol. Peak 7 showed a molecular ion at *m/z* 607 and the loss of 322 amu, corresponding to the glucuronyl-rhamnosyl residue suggesting the kaempferol glucuronyl-rhamnoside. Peak 10 exhibited a molecular ion of 461, and a MS^2^ fragment at *m/z* 285 from the loss of 176 amu (glucuronyl residue), a fragment pattern common to kaempferol glucuronide.

UV spectra for peaks 5 and 8 revealed a maximum near 260 nm and an absorption peak around 350 nm. These characteristics are consistent with UV spectra profiles from quercetin derivatives. Peak 5 exhibited a molecular ion at *m/z* 623, originating MS^2^ fragments of 301 (loss of 322 amu) and 459 (loss of 164 amu), corresponding to the loss of glucuronyl-rhamnoside and rhamnoside residues, respectively. Consequently, this compound was tentatively identified as quercetin glucuronyl-rhamnoside. Peak 8 presented a molecular ion of 477, leading to a fragment at *m/z* 301 (loss of 176 amu) at the MS^2^ spectrum, corresponding to the loss of a glucuronyl residue. This result suggests that this quercetin derivative is a quercetin glucuronide.

Peaks 6, 9, and 11 were identified based on their UV spectra, presenting absorption maximum at 254 nm and between 360–380 nm, consistent with that of ellagic acid and its derivatives [25,26]. The MS spectra of peak 9 has a molecular ion at *m/z* 301 and fragments at *m/z* 283, 257, and 229, suggesting the presence of ellagic acid. This structure was confirmed using a commercial standard by comparing retention time, UV, and MS fragmentation pattern. Mass spectra of peak 11 exhibited a molecular ion at *m/z* 461 and MS*2* signals at 315 (loss of 146 amu) and 446 (loss of 15 amu) resulting from the loss of deoxyhexose and methyl groups, respectively, which could be interpreted as a methyl ellagic acid rhamnoside structure from Liberal et al. [5].

### 3.2. Evaluation of Tyrosinase Inhibitory Activity of the Extract

Tyrosinase inhibitors have been used in whitening skin care products because tyrosinase is a key enzyme in melanin formation. The extract obtained from the leaves of *F. vesca* extract was tested for its ability to inhibit tyrosinase and consequently evaluate its antimelanogenic potential. The extract exhibited inhibitory activity for the enzyme, with an IC50 of 238.10 ± 15.51 µg/mL, compared to the IC50 value obtained for arbutin of 193.84 ± 14.15 µg/mL. Ellagitannins, ellagic acid, and its derivatives present in the extract could be responsible for this effect since these compounds are known to inhibit tyrosinase, mainly by cooper quelation [27,28]. Other effects attributed to these phenolic compounds, including the suppression of enzyme expression and antioxidant activity, could also contribute to the decrease in melanin formation [11]. These results indicate that the extract may have potential in reducing melanin formation and consequently could be used as an active ingredient in whitening skin care products. Considering that the arbutin (used as control) is safely used in cosmetics in concentrations of 2%, the same concentration was used for incorporating the *F. vesca* extract in the hydrogel formulation [29].

### 3.3. F. vesca Based Hydrogel Stability Studies

The results of stability analysis in different conditions (room temperature, light; room temperature, dark; 5–9 °C; 40 °C with 75% RH) did not reveal significant changes. Concerning the organoleptic analysis, the changes verified at the end of seven days remained until the end of the assay (28 days). All samples kept at room temperature maintained the initial color, smell, and aspect, i.e., greenish brown and homogeneous. The hydrogel conserved in the absence of light was dark-green, retaining the initial characteristics regarding color and appearance. The sample subjected to the cycles of sunlight developed a brownish-green color, due to the oxidation of the compounds. The sample stored at 40 °C darkened over time and its consistency became more fluid. The initial color changed from light green to dark green.

Concerning pH results, the initial pH of 5.38 decreased for all samples, with the highest decrease occurring during the first week of tests. Nevertheless, all samples remained acidic, being suitable for skin application, since the pH of skin products should be in the range 4.5–6.5 [17]. Sample S4, stored at 40 °C, showed the highest pH variation, from 5.38 to 4.88, while S3, stored in the cold, showed less variable pH values (5.38–5.30). Samples S1 and S2, stored at room temperature in the light and in the dark, respectively, showed a decrease in pH values from 5.38 to 5.10 and 5.15, respectively. The surface of the skin has a naturally acidic pH, which is extremely important in regulating protective functions, maintaining the lipid barrier homeostasis and the integrity of the *stratum corneum* [30]. Acidification of the *stratum corneum* also has an antimicrobial function since acidic pH inhibits colonization by pathogenic bacteria [31]. The use of cosmetic products with pH values that mimic the natural pH of the skin minimizes the disturbances to the conditions of development of the resident microflora [18].

The changes in viscosity results are in accordance with previous results. The values varied from 12,000 to 31,000 mPa s. These variations were expected since, normally, viscosity is directly influenced by temperature variations [32]. Sample S1, stored at room temperature in the light, showed an evident degradation of the compounds, as revealed by organoleptic analysis, as well as a decrease in viscosity. Sample S2, also kept at room temperature but protected from sunlight, maintained viscosity values at around 20,000 mPa s. The greatest variation over the 28 days was observed for the sample stored in the fridge (S3), which viscosity increased from the initial 20,000 to 30,000 mPa s. The storage conditions of sample S4 led to a decrease in viscosity due to high temperature, resulting in the formation of weaker hydrogels, consistent with that described in the literature by Wang et al. [33]. These results, as well as the ones obtained in the organoleptic analysis, where the appearance and color of the formulation changed, strongly suggest that in this sample the properties of the extract were altered. Elevated temperatures can accelerate chemical reactions, altering the activity of the components, viscosity, appearance, color, formulation odor and flavor, and leading to the degradation of phenolic compounds, since these are sensitive to hydrolysis [32].

### 3.4. Texture Profile Analysis of the Formulation

Texture properties are an important parameter for optimization of topical formulations since they influence the applicability of the hydrogel at the administration site [34]. The mechanical parameters obtained for the four samples are presented in Table 2.

Hardness and compressibility of a formulation are related to the ease of extraction of the hydrogel from the container and its application; if these values are low, it is ensured that the hydrogel is easily applicable to the skin [19,34]. Correlations were observed between increased formulation viscosity and increased product hardness and compressibility [35].

Concerning texture profile, samples S1 and S3 exhibited similar behaviors, although the viscosities were not comparable. This can be explained by the oxidation of the active compounds and degradation of the polymer in sample S1, after its exposure to light.

Sample S2 is the equivalent of the initial hydrogel and showed no significant variations. The sample with higher viscosity (S3) presented values of hardness and compressibility predictably higher (approximately double) than at day 0.

In sample S4, the decrease in these values is related to an increase in temperature, which was expected, given its lower viscosity relatively to other samples. However, during the time for preparation (removal from greenhouse and preparation of the sample for measurements), this sample was at room temperature and regained some of its original consistency, which explains the results obtained.

Adhesiveness is the work required to overcome the attractive forces between the surface of the sample and the surface of the probe and is related to hydrogel retention in the skin and bioadhesion [19,20,34].

Table 2 shows the variation of adhesiveness, and it can be observed that the values are consistent with those obtained for compressibility and hardness, with the largest determined negative areas corresponding to samples S1 and S3. The greater negative area obtained for sample 3, of higher viscosity, is also due to the fact that the adhesiveness is a parameter dependent on the polymer concentration [34].

Cohesiveness is defined as the work required to deform the hydrogel during the downward movement of the probe, and it provides information about the structural reforming of the hydrogel after application; in general, a high value is associated with a total structural recovery. The data obtained on the cohesion of the HEC hydrogel are relatively a little variable and in agreement with those described in the literature [19,20,35].

Elasticity is defined as the rate at which the deformed sample returns to its original condition after the removal of the deforming force [19]. Low values reveal that the formulation is more susceptible to structural deformations [19,20,35]. Elasticity proved to be the most consistent and least variable parameter between the four samples, and at different temperatures. The values approximate the unit, which leads to the conclusion that all four samples easily recover their original shape after deformation.

### 3.5. Total Tannin Content in Extract and Hydrogel Formulation

The results of the assay revealed that the extract contained 11.79% tannins, corresponding to 3.35% in the plant, while the *F. vesca* based hydrogel contained 0.5% tannins. The value obtained for the extract is similar to previously obtained results (2.3%), determined using the same experimental method [36]. In all studies the polyphenol content showed a positive correlation with the antioxidant potential of the extracts [4,37]. The ellagitannins were predominant in the extract and could be responsible for the tyrosinase inhibition. Previous studies reported the antimelanogenic activity of these compounds [11,27].

### 3.6. Cytotoxicity Studies

**Figure 2:**
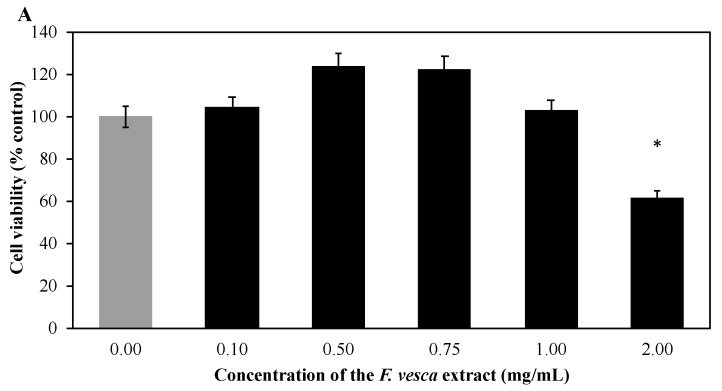

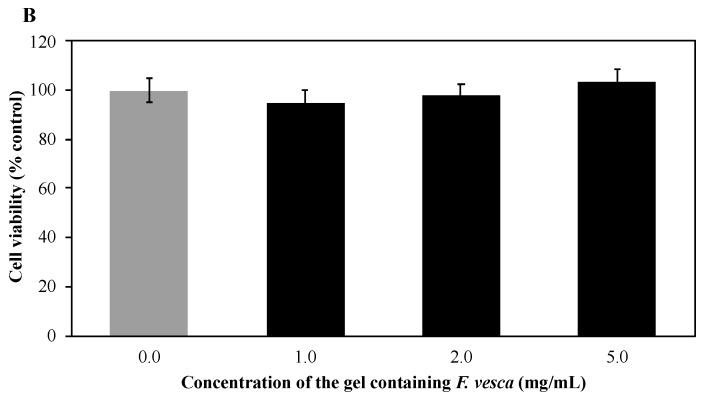
(**A**) Effect of *F. vesca* extract and (**B**) *F. vesca* based hydrogel on HaCaT cells viability. Cells were treated with different concentrations of extract or hydrogel formulation for 24 h and the cell viability was assessed by the MTT reduction assay. The data are presented as the Mean ± SEM of five independent experiments run in quadruplicate and expressed as percentage of control cells (100%). * *p*-value is significant compared to the control group (*p* < 0.05).

The cytotoxicities of the extract and the hydrogel formulation were evaluated in HaCaT cells, after incubating the cell cultures with different concentrations of the extract and hydrogel for 24 h, using the MTT assay (Figure 2). 

The analysis of the results showed that the extract at a concentration of 2 mg/mL reduced the viability of HaCaT cells, thus being cytotoxic at this concentration. Some flavonoids—kaempferol, quercetin, isorhamnetin—have been described in the literature as inhibitors of cell growth. Thus, the high concentrations of kaempferol and quercetin derivatives in the extract may be responsible for the observed reduction of cell viability [11,26].

The hydrogel clearly demonstrates no toxicity at all concentrations tested (Figure 2B). These results reinforced the results obtained by Gardner and McGuffin and available in the European Union herbal monograph on *Fragaria vesca* L., Fragaria viridis West., Fragaria moschata West., *Fragaria ananassa* (West.) Duchesne ex Rozier, foliu (EMA/HMPC/432276/2015). Gardner and McGuffin concluded that *F. vesca* can be considered as safe (Class 1) and no clinically-relevant interactions are expected (Class A).

Considering that the hydrogel formulation contains ethanol as a preservative and that ethanol may have cytotoxic effects—slower cell growth, decreased cell proliferation, and accelerated apoptosis [38]—the effect of ethanol on the viability of HaCaT cells was also evaluated. Ethanol showed no toxicity at the concentrations used. Thus, since the formulation did not show deleterious effects on keratinocytes, it was considered a promising ingredient for topical application.

### 3.7. Reactive Oxygen Species (ROS) Production Measurement

**Figure 3:**
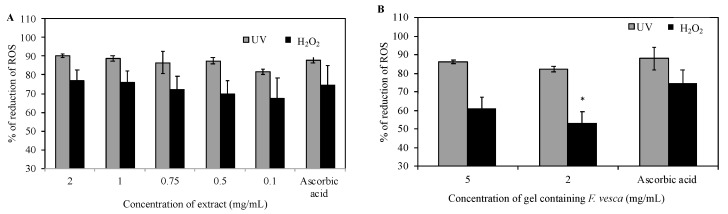
(**A**) Effect of *F. vesca* extract and (**B**) *F. vesca*-based hydrogel on the percentage of reduction of ROS production in HaCaT cell cultures in the presence of H_2_O_2_ during 1 h, or when the cells are UVB-irradiated for 15 min. Ascorbic acid (AA) was used as a positive control. The statistical comparison with the positive control is also shown (* *p* < 0.05) (Mean ± SD, *n* = 6).

As shown in Figure 3, the *F. vesca* extract and the *F. vesca* based hydrogel showed ROS scavenging activity when exposed to UV radiation. The *F. vesca* extract (1 mg/mL), the *F. vesca* based hydrogel (5 mg/mL), and the ascorbid acid led to 88.7 ± 1.5%, 86.2 ± 1.7%, and 88.0 ± 2.8% of ROS reduction, respectively (*p* < 0.05). In the assay using H_2_O_2_, *F. vesca* extract (1 mg/mL), the *F. vesca* based hydrogel (5 mg/mL), and the ascorbid acid led to 75.9 ± 6.3%, 61.1 ± 10.3%, and 74.5 ± 3.3% of ROS reduction, respectively (*p* < 0.05). In this test, *F. vesca* extract and the *F. vesca* based hydrogel revealed an excellent antiradical activity. In addition, these results revealed that the bioactive *F. vesca* extract retain the antioxidant activity, even when incorporated in the complex matrix of a hydrogel. Previous studies reported identical results [39,40]. Marques et al. [39] evaluated the antioxidant effect of *Cynara scolymus* L. extracts incorporated into a hydrogel, and observed that the topical hydrogel maintained almost entirely the antioxidant activity exhibited by the extract. Studies performed by Barreira et al. [23,24] revealed a similar antioxidant activity of ethanolic and aqueous *Crataegus monogyna* extracts alone and incorporated in hydrogels, suggesting that the complex matrix of hydrogel does not interfere with the antioxidant activity of the extracts.

As shown in Figure 3, the hydrogel matrix of the topical formulations does not retain the bioactive compounds present in the *F. vesca* extract, and shows identical activity when compared to *F. vesca* extract.

### 3.8. F. vesca Based Hydrogel Safety Tests

The simple patch test carried out on 10 volunteers showed a good compatibility of the product with the skin, with no irritant reactions observed. Likewise, repeated applications (HRIPT assay) of the formulation in 50 volunteers did not cause irritant reactions, neither in the induction phase nor in the challenge phase. Compared with the single patch test, the HRIPT is the relevant study, since it is a set of successive patch tests and therefore a much more complete and robust in vivo security test. The in vivo results are in agreement with cell viability assay results obtained with the hydrogel. Taken together, these results show that the hydrogel formulation containing *F. vesca* extract is suitable for topical application.

### 3.9. Antioxidant Efficacy

**Figure 4:**
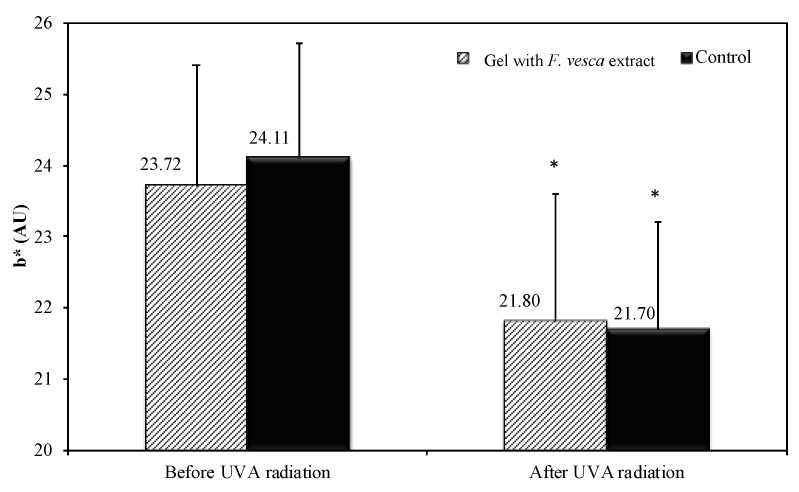
The b* parameter evolution before and after UVA irradiation (Mean ± SD, *n* = 21). Moreover, the statistical comparison against D0 is shown (* *p* < 0.05).

To evaluate the in vivo antioxidant activity, an analysis of the skin color was performed and compared to the first day of topical application of the products (D0) was performed. The color variation (b* parameter) in the control arm (10.0%) was significantly higher than the variation in the arm treated with hydrogel formulation containing *F. vesca* (8.1%). The difference between the variation was 1.9%. Figure 4 shows the values of b* parameter before and after UVA irradiation, in the control arm, and in the arm treated with hydrogel with *F. vesca* extract.

**Figure 5:**
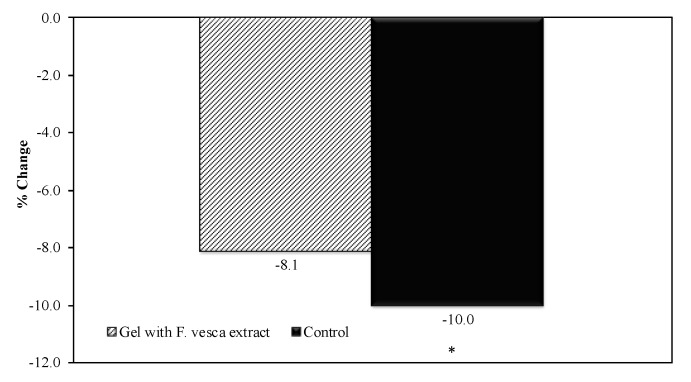
The b* parameter % change before and after UVA irradiation (Mean ± SD, *n* = 21). Moreover, the statistical comparison against the D0 is shown (* *p* < 0.05).

To evaluate the true changes in the b* parameter, a transformation relative to D0 was performed. The results are summarized in Figure 5.

This result shows that the product has the ability to decrease the oxidation of β carotene under UVA irradiation thus it can be considered an antioxidant. Previous in vitro and in vivo studies with topical O/W emulsions and hydrogels containing the *C. scolymus* extract, also revealed an antioxidant effect [39].

## 4. Conclusions

The optimized *F. vesca* extract obtained in this study was rich in elagitannins and showed anti-melanogenic potential, being a promising antioxidant cosmetic ingredient.

A physicochemical characterization of the hydrogel formulations containing the *F. vesca* extract was performed and all formulations showed acidic pH values and appropriate viscosity values, hence they were considered suitable for skin application. The formulation kept at room temperature and protected from sunlight presented the highest stability.

The cytotoxicity assays in HaCaT cells showed that *F. vesca* based hydrogels were also suitable for topical application. Furthermore, the formulations revealed suitable ROS scavenging properties in the presence of H_2_O_2_ after UVB radiation exposure.

The HRIPT study performed in human volunteers showed very good skin compatibility for the 2% *F. vesca* extract-based hydrogel, which was considered dermatologically safe.

As a conclusion, we developed a hydrogel formulation containing 2% of *F. vesca* extract and have shown, for the first time, its suitability for topical application, as well as its potential as an antioxidant cosmetic ingredient.

## Figures and Tables

**Table 1:** Qualitative and quantitative composition of *F. vesca* based hydrogel.

Ingredients	Quantitative Composition (%, *w/w*)
*F. vesca* Extract	2.0
Hydroxyethylcellulose (HEC)	2.5
Ethanol (96%)	22.5
Purified Water	73.0

**Table 2:** Variation of texturometer parameters (hardness, compressibility, adhesiveness, cohesiveness, elasticity) from day 0 to day 28 for different conditions (Mean ± SD, *n* = 3).

Time (Days)	Hardness (g)	Compressibility (g.sec)	Adhesiveness (g.sec)	Cohesiveness	Elasticity
D0	19.201 ± 0.65	23.808 ± 0.4	−23.953 ± 0.54	0.853 ±0.04	0.998 ± 0.08
D28 (S1)	37.515 ± 0.47	47.118 ± 0.25	−36.157 ± 0.47	0.725 ± 0.06	0.918 ± 0.02
D28 (S2)	21.865 ± 0.24	22.504 ± 0.42	−24.552 ± 0.61	0.973 ± 0.04	0.941 ± 0.03
D28 (S3)	40.289 ± 0.27	42.134 ± 0.4	−40.967 ± 0.28	0.877 ± 0.03	0.955 ± 0.05
D28 (S4)	22.309 ± 0.39	23.367 ± 0.64	−30.453 ± 0.22	1.038 ± 0.04	0.995 ± 0.07
